# Development of a core evaluation framework of value-added medicines: report 1 on methodology and findings

**DOI:** 10.1186/s12962-021-00311-6

**Published:** 2021-08-31

**Authors:** Zsuzsanna Ida Petykó, Zoltán Kaló, Jaime Espin, Kateřina Podrazilová, Tomáš Tesař, Nikos Maniadakis, Frank-Ulrich Fricke, András Inotai

**Affiliations:** 1 Center for Health Technology Assessment, Semmelweis University, Üllői rd. 25, Budapest, 1085 Hungary; 2 Syreon Research Institute, Mexikói str. 65/A, Budapest, 1142 Hungary; 3 Andalusian School of Public Health, Granada, Spain; 4 Association of Health Insurance Companies, Prague, Czech Republic; 5 Department of Organisation and Management of Pharmacy, Faculty of Pharmacy, Comenius University in Bratislava, Bratislava, Slovakia; 6 Department of Public Health Policies, Sector of Health Systems and Policy, School of Public Health, University of West Attica, Athens, Greece; 7 Technische Hochschule Nürnberg, Nürnberg, Germany

**Keywords:** Expert panel, Generic price erosion, Incremental innovation, Value-added medicines, Repurposed medicine, Value assessment framework, Value domain, Value proposition

## Abstract

**Background:**

Medicines that are based on known molecules and are further developed to address healthcare needs and deliver relevant improvement for patients, healthcare professionals and/or payers are called value-added medicines (VAMs). The evaluation process of VAMs is heterogeneous across countries, and it has been primarily designed for originator pharmaceuticals with confirmatory evidence collected alongside pivotal clinical trials. There is a mismatch between evidence requirements by public decision-makers and evidence generated by manufacturers of VAMs. Our objective was to develop a core evaluation framework for VAMs.

**Methods:**

Potential benefits offered by VAMs were collected through a systematic literature review and allocated to separate domains in an iterative process. The draft list of domains and their applicability were validated during two consecutive virtual workshops by health policy experts representing countries with different economic statuses, geographical and decision-making contexts.

**Results:**

Based on 158 extracted studies, the final consensus on the evaluation framework resulted in 11 value domains in 5 main clusters, including unmet medical needs, health gain (measured by health care professionals), patient-reported outcomes, burden on households, and burden on the health care system.

**Conclusions:**

The proposed framework could reduce the heterogeneity in value assessment processes across countries and create incentives for manufacturers to invest in incremental innovation. However, some domains may not be equally relevant or accepted in all countries, therefore the core framework needs thorough adaptation in specific jurisdictions.

**Supplementary Information:**

The online version contains supplementary material available at 10.1186/s12962-021-00311-6.

## Background

To ensure the sustainability of health care financing, international guidelines recommend the first-line use of off-patent medicines in major diseases [1, 2]. In those diseases, where recent pharmaceutical innovation has delivered significant added therapeutic value compared to the currently used standard therapy, utilizing available innovative pharmaceuticals is justifiable. However, the recent coronavirus (SARS‐CoV‐2/COVID-19) pandemic has highlighted the importance of timeliness in pharmaceutical innovation, which does not necessarily mean revolutionary (or disruptive) innovation [3–5]. Incremental (also known as continuous or evolutionary) innovation of medical therapies means the process of gradually improving existing medicines, devices and services [6–8]. Incremental innovation of existing medicines can provide an affordable answer to unmet medical needs due to shorter development time and lower research and development costs compared to the development of originator medicines.

Currently, there is no consensus on whether *drug repurposing* should refer only to developing new indications of established medicines or it should cover all categories of incremental (evolutionary) pharmaceutical innovation [9]. Therefore, *value-added medicine* (VAM) has been used in recent publications as a broad collective term, referring to all different types of medicines further developed by repositioning, reformulating or combining known molecules that address health care needs and deliver relevant improvements for patients, health care professionals and/or payers [10–13]. *Repositioning* refers to finding new indications or applying a medicine in a new patient population [14, 15]. *Reformulation* is a process in which the pharmaceutical formulation of a product is modified to gain new value attributes. *Combination* of established compounds in one product (e.g., fixed-dose combination) or medicines combined with medical devices (e.g., self-injector) and services (e.g., mobile application) including digital technologies may deliver additional value to patients and health care professionals [16]. Some examples of VAMs created through the main repurposing models are presented in Additional file 1: Table S1 [17].

However, several barriers prevent society from maximizing the benefits of continuous innovation delivered by VAMs, as reported by our research group [18] and others [8]. Most importantly, current evaluation frameworks used for pricing and reimbursement decisions have been primarily designed for originator pharmaceuticals with evidence on benefits collected mainly in pivotal clinical trials. As large-scale clinical trials are not required for market authorization in most cases of VAMs, the evidence base of their differential value is less abundant. Additionally, several value propositions delivered by repurposing (e.g., improved adherence, opportunity for self- and home administration) are based on surrogate outcomes, which benefits captured in traditional value elements (i.e., improved quality-adjusted life years, survival or reduction of direct health care costs) can be validated only in costly and lengthy prospective trials or with real-world evidence [19]. Despite recent efforts to standardize health technology assessment (HTA) activities for innovative health technologies through EUnetHTA in the European Union, implementing a global evidence generation strategy to support the introduction of new VAMs is still difficult for market authorization holders [20]. The evaluation process of VAMs remains heterogeneous across countries. In most cases, this phenomenon is partly due to the mismatch between evidence requirements by public decision-makers and evidence generated by manufacturers and other stakeholders.

It is becoming obvious that several medicines could be repurposed following patent expiry and that these developments could deliver great benefits for society worldwide [18]. If policy-makers want to encourage this activity, value creation through repurposing pharmaceuticals needs to be rewarded appropriately. Improved clarity on value assessment frameworks and consistency across different jurisdictions can provide a signal to developers. Our objective was to develop a core evaluation framework for VAMs, that could serve as a starting point for (future) national adaptations in different countries. To the best of our knowledge, this is the first study that was conducted to map potential attributes of incrementally innovated medicines. The attributes identified primarily from a systematic literature review (SLR) were incorporated into a value assessment framework, which was then validated by a panel of international health policy experts, who also assessed its adaptability to various decision-making contexts.

The findings and implications of this research are summarized in two separate reports; this article (Report 1) summarizes the methodology and results of framework development and briefly discusses the most relevant high-level policy consequences of this work. Report 2 is a separate publication that contemplates key policy implications of our research, namely the adaptation of the framework to different decision-making contexts and the complexity of evidence generation implied by the framework [21].

## Methods

### Literature review and data extraction

To identify all relevant articles and available materials and to provide a rigorous scientific base for a comprehensive list of value propositions offered by VAMs, an SLR was conducted, supplemented by a targeted literature review (TLR). The SLR search syntax was designed to collect value propositions based on current examples with a scope to allow the framework to capture value delivered by potential future innovations as well. The search query was built up from a combination of four sets of keywords. The first two sets comprised of synonyms of *value-added* and *medicine* to identify compound words for VAMs that are composed according to this scheme. The third set was created to include those complex synonyms where the search terms could not be divided into phrases collected by sets #1 and #2, as described above. Therefore, set #3 collected terms analogous to the expression *value-added medicine*. As the aim of the research was to identify all potential value propositions, the fourth set contained synonym keywords on *benefits* and *value*. Since existing frameworks on the public domain were expected to report not only a list of potentially applicable value domains but also definitions or guides to measurement, set #4 also collected keywords on *value frameworks* and *multi-criteria decision analysis (MCDA)*. The four sets were combined with Boolean operators (AND/OR) as follows: (((#1 AND #2) OR #3) AND #4) (Additional file 1: Table S2).

The search query was implemented in the Medline database through Pubmed on 21 June 2019. No restrictions on the publication date or article type were applied; however, only English-language articles were considered eligible during the screening process. Hits identified by the search syntax were further processed with the EndNote X6 software. Consideration of eligibility for the title and abstract screening, followed by full-text screening, was based on predefined explicit inclusion and exclusion criteria. Both screening steps were performed by two researchers independently and disagreements between reviewers regarding inclusion and exclusion were resolved by a principal researcher not involved in the primary screening processes.

The following exclusion criteria were used during the title and abstract screening: (1.1) no English abstract available, (1.2) English abstract of non-English full-text paper, (1.3) animal, in vitro study, in silico study or other preclinical studies; review article only mentioning repurposing but unlikely that even its full-text would contain any potential value propositions for VAMs, (1.4) abstract explicitly not related to the research topic (i.e., value assessment of VAMs), (1.5) value proposition from low-income countries without an established health care system.

All articles not excluded during the title and abstract screening phase were considered for full-text screening. The exclusion criteria for the full-text screening were the following: (2.1) no English abstract available, (2.2) non-English full text, (2.3) animal or in vitro, in silico or other preclinical studies, (2.4) full text not related to the research question, (2.5) value proposition from a low-income country without an established health care system, (2.6) full text with no value propositions reported, (2.7) duplicate publication, (2.8) full text not accessible. Publications not excluded during the full-text screening were eligible for data extraction. A snowball search was also performed on extracted articles to identify further relevant studies among their references.

Parallel to the SLR, a TLR was performed to identify additional documents from grey literature sources, including conference materials and reports recommended by member organizations of Medicines for Europe (MFE), an umbrella organization representing European pharmaceutical companies manufacturing generic, biosimilar and value-added medicines. Materials considered eligible for data extraction originated from (1) the SLR, (2) the snowball method and (3) grey literature materials identified by the TLR.

A predefined data extraction spreadsheet was developed in Microsoft Excel to standardize data collection among researchers. Data extracted by a researcher was double-checked by another one and disagreements were resolved by a principal researcher. The following data categories were extracted from the included studies:

1. General study data including author, publication year, title, study type, study objectives, study conclusions (if relevant), country of origin;
2. Active compound-related data including the name of active compound(s), the applied repurposing model (repositioning, reformulation or combination) and disease area;
3. Value proposition-related data including the name, description, definition, information on the measurement of potential value propositions and example products (if mentioned).

A value proposition was considered to be a word, term or complex expression describing any health, monetary, social, etc. benefit(s) delivered by a VAM. Data extractors employed two distinct ways to capture all potential value attributes (even ones with seemingly minor relevance or without any existing reference medicine): a standard and an alternative method. During the standard method, reviewers extracted value propositions literally, with the precise wording, as it was reported by the publication. On the other hand, the alternative method was used to create value propositions by the data extractors inspired by the original text of the article. The method of extraction was required to be indicated next to all value propositions in the data extraction spreadsheet by the reviewers.

### *Framework *development and validation

#### Transforming value propositions into value domains

The data extraction was followed by the development of potential value domains for an evaluation framework by grouping all the extracted value propositions. Therefore, the SLR and TLR data extraction spreadsheets were reviewed row by row, duplications and synonyms of terms were removed, and individual value propositions were grouped under draft umbrella terms for value domains. This process was also performed by two researchers.

#### Internal development and validation of draft domains

Subsequently, the initial list of value domains, along with the related value propositions, were reviewed by a group of experts (with experience on developing and adapting MCDAs or value frameworks and with a background in HTA, academics or patient advocacy) in multiple iterative rounds. The main goal of the development/internal validation was to minimize overlaps between the proposed domains and to provide an early feasibility assessment of using the draft domains in various decision-making contexts [22, 23]. Draft value domains were further merged under domain clusters, each cluster containing 2–3 domains. Following this internal validation, the Value Added Medicines sector group of Medicines for Europe had the opportunity to comment on the draft framework.

#### External validation of draft domains

To increase the validity of the draft evaluation framework, nine health policy experts and decision-makers with a thorough understanding of different decision-making contexts from selected Western European (United Kingdom, Germany), Southern European (Spain, Greece), Northern European (Denmark), and Central & Eastern European (Czech Republic, Slovakia, Slovenia, Lithuania) countries were invited to an external validation process. The selection criteria of experts were familiarity with the evaluation and reimbursement process of pharmaceuticals in different health systems. Efforts were made to achieve a balanced distribution of participants regarding their country of residence and stakeholder perspective. Therefore, experts represented countries with different economic status (i.e., high vs. low income), geographical location, HTA capacities and systems (i.e., primary reliance on relative effectiveness or cost-utility analyses), also different stakeholder groups (i.e., healthcare payers, HTA professionals, health policy experts) and sectors (public payer and governmental institute, private payer and academic). As a consequence of the COVID-19 pandemic, the originally planned face-to-face validation meeting was replaced by two consecutive half-day virtual meetings, where participants were encouraged to share their insights and provide recommendations for further improvement. During the first virtual meeting, researchers presented the background, methodology and development of the draft evaluation framework in detail and facilitated a short, moderated discussion for prompt questions. Between the two workshops, participants were asked to fill in a standardized feedback form on (1) the structure of the proposed evaluation framework, (2) the name, definition, full list of value propositions (as a word cloud) allocated to each individual value domain, (3) any issues regarding the evidence generation for VAMs and (4) applicability of the proposed value framework in different decision-making contexts.

After processing feedback from participants, researchers amended the draft evaluation framework for the second virtual meeting and prepared a set of illustrative test cases for further validation. This virtual meeting aimed to reflect on, discuss and create consensus regarding feedback on the draft framework through discussion and anonymous voting. Both virtual meetings took place in June 2020, with two weeks in-between. One representative of MFE participated in the meetings as a silent observer.

## Results

**Fig. 1:**
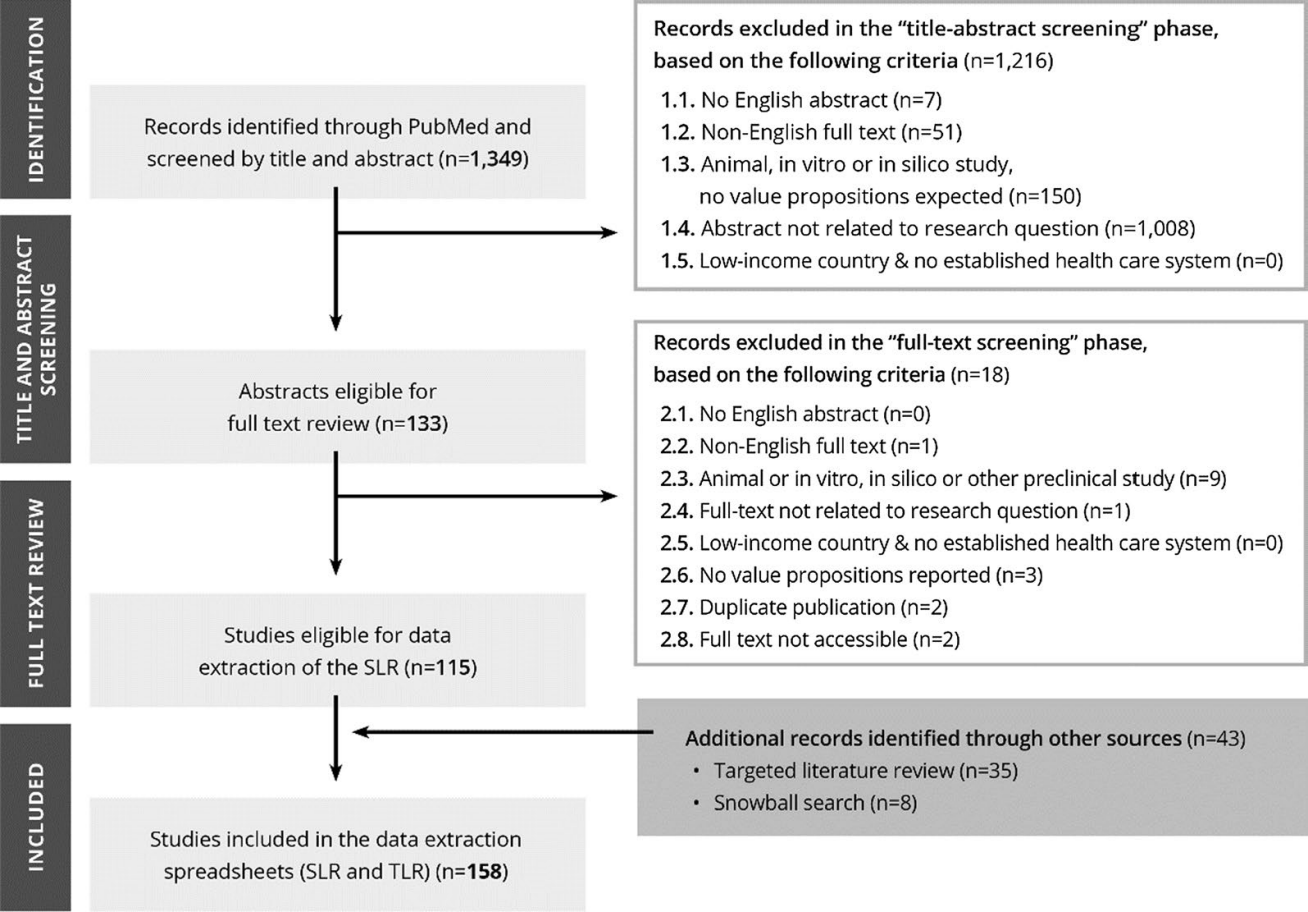
PRISMA flow diagram of the literature review. *SLR* systematic literature review, *TLR* targeted literature review

The search syntax of the SLR resulted in 1349 hits, of which—after title, abstract and full-text screening—115 full-text articles were found eligible for inclusion. The snowball search provided 8 additional articles, and 35 additional documents were included through the TLR. In total, 158 studies were extracted. The complete flowchart of the literature review based on the principles of the PRISMA statement is presented in Fig. 1 [24].

The most frequent study type among the included publications were non-systematic reviews (83%) and SLRs (6%). Contradictory to our expectations, only 3 MCDA frameworks with potentially relevant value domains were identified and no additional value frameworks were published in the scope of the search. The included studies referenced approximately 350 different active compounds in relation to VAMs. Regarding the type of repurposing, most of the articles discussed repositioning of an established active compound (72%), while reformulation and combination of medicines occurred only in 16% and 12% of the mentioned repurposing, respectively. Most of the articles provided sufficient data on active compounds, type of repurposing models and disease area. However, only one-third of the included publications presented a clear definition for the proposed values; moreover, measurement scales and scoring functions of such value propositions were rarely reported.

**Fig. 2:**
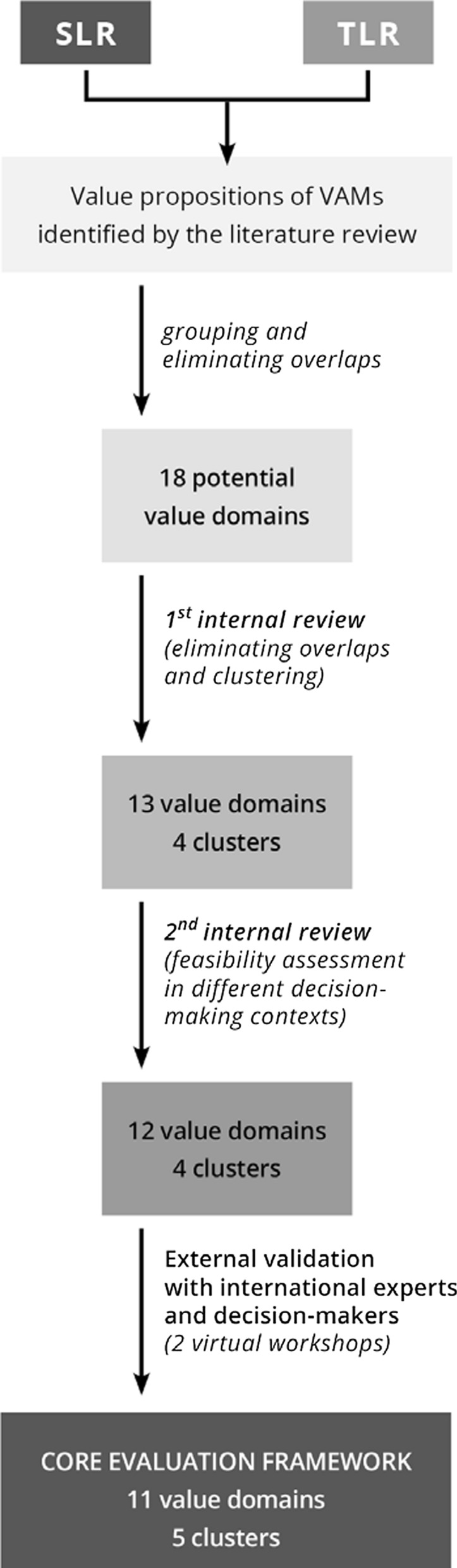
Development process of the evaluation framework for value-added medicines. *SLR* systematic literature review, *TLR* targeted literature review, *VAM* value-added medicines

Altogether 907 (668 from the SLR and 239 from the TLR) specific value propositions were identified from the literature before eliminating repetitions, synonyms and overlapping terms. The initial step of transforming value propositions into preliminary value domains resulted in 18 draft domains. The iterative internal review reduced the number of individual value domains to 12, under 4 main clusters. Following the external validation and anonymous voting, the final consensus on the evaluation framework resulted in 11 individual value domains grouped under 5 clusters. The core evaluation framework's course of development is presented in Fig. 2.

The 11 value domains are the following: (1) Extending treatment options in a new indication with unmet medical need, (2) Individual needs/special needs of patient (sub)population, (3) Efficacy/Effectiveness, (4) Patient safety and tolerability, (5) Patient experience related to the therapy, (6) Adherence and Persistence, (7) Quality of life, (8) Patient's economic burden, (9) Economic and health burden on informal caregiver, (10) Health care resource utilization, costs or efficiency and (11) Technological improvement with logistical considerations. Final clusters, domains and related definitions of the evaluation framework are presented in Table 1. Illustrative test cases (in Additional file 1: Table S3) and non-exhaustive, illustrative value propositions (in Additional file 1: Table S4) are presented as supplementary material.

**Table 1:** The core evaluation framework for value-added medicines

Cluster name	Domain name	Definition
Unmet medical need	Extending treatment options in new indication with unmet medical need	Reduction of the unmet medical need for patients in a new indication due to additional therapeutical value
	Individual needs/special needs of patient (sub)population	Reduction of the unmet medical need in patients with special needs in the original indication (e.g., treatment resistant patients, vulnerable patients, etc.)
Health gain (measured by health care professionals)	Efficacy/Effectiveness	Improved clinical outcomes of a pharmaceutical treatment (e.g., extending survival, stabilizing disease, improving treatment response, etc.) in trial and real-world settings
	Patient safety and tolerability	Improved safety and/or tolerability of the pharmaceutical treatment
Patient Reported Outcomes	Patient experience related to the therapy	Improved patient's satisfaction, acceptance, convenience with the pharmaceutical therapy
	Adherence and persistence	Improved adherence and/or persistence of patients with the prescription guidelines (including duration, timing, dosage and frequency of medication use)
	Quality of life	Improved health-related quality of life reported by patients
Burden on households	Patient's economic burden	Improved productivity of patients and/or reduced health and non-health care resource use (such as travel time) covered by patients
	Economic and health burden on informal caregiver	Improved quality of life of family caregivers and/or reduced financial or non-financial burden on households
Burden on health care system	Health care resource utilization, costs or efficiency	Reduced utilization of health care cost and/or resources
	Technological improvement with logistical considerations	Improved stability and/or shelf life of pharmaceuticals through technological improvement

*VAM* value-added medicine

## Discussion

With regards to R&D pathways, regulatory incentives, expected evidence base for differential value and the potential return on investment, VAMs represent a special category of pharmaceuticals, being neither purely generics nor purely originator medicines [25]. Nevertheless, they are not fully recognized as such by several stakeholders. In the evaluation process to support pricing and reimbursement decisions, VAMs are dominantly approached from two perspectives: either their benefits are assessed similarly to benefits of originator medicines; thus the same level of evidence is expected, or they are perceived as generics and cost-minimization analysis (focusing only on the potential cost-savings to other medicines with the same active product ingredient) is applied in determining their value. Hence, the differential value of VAMs may not be fully recognized in these approaches, which consequentially diminishes incentives for investment into the incremental innovation of these pharmaceuticals resulting in a loss of opportunity for society. Since the regulatory pathway for continuous innovation of established medicines differs from both revolutionary (or disruptive) innovation of originator medicines and launching generics after patent expiry, the evaluation process of VAMs should take these differences into account in order to support well-founded policy decisions. Appropriate value assessment frameworks should be more receptive to alternative methods of evidence generation (e.g., real-world evidence, outcome guarantee, coverage with evidence development) or should be extended with additional value domains that are highly relevant for VAMs (e.g., patient adherence, patient experience, technological improvements). Such an extension of traditional evaluation frameworks with new value domains or new evidence types would also be beneficial for other types of special technologies, such as digital health solutions, combined technologies or gene therapies.

This review aimed to identify value propositions of VAMs, not only from existing cases but from possible future innovations as well. This intention is even reflected by the search strategy: instead of collecting evidence only from clinical trials and highly cited case studies, our review scope allowed for the inclusion of a much broader spectrum of references. The high proportion of review-type articles (83%) among the included studies confirmed that the chosen search strategy was fit-for-purpose.

The majority of the proposed value domains for VAMs overlap with the assessment criteria of originator medicines, which could be explained by the fact that manufacturers of originator medicines and manufacturers of VAMs aim to address similar unmet medical needs. Still, specific domains (i.e., extending treatment options in new indication with unmet medical need, improved adherence and persistence, technological improvement with logistical considerations) are far more relevant to VAMs compared with originator medicines. For example, extended shelf-life is a typical benefit of VAMs, which is less applicable for original pharmaceuticals.

In addition, the proposed evaluation framework of VAMs facilitates a patient-centered value assessment approach, since 5 out of the 11 value domains (Patient experience related to the therapy, Adherence and Persistence, Quality of life, Patient's economic burden, Economic and health burden on informal caregiver) are reflecting the perspective of patients and their caregivers in the therapeutic process [26]. The overlaps between this VAM-specific evaluation framework and current HTA procedures may ease the framework adaptation in different jurisdictions while promoting a shift towards a more patient-centered value assessment approach for reimbursement decisions [27, 28]. Whilst patient centricity has been in the focus of HTA bodies in many countries, recommendation for explicit criteria in a core evaluation framework can facilitate the evidence generation in patient centric value domains by multinational manufacturers in parallel with improving the degree of patient centricity and reducing the heterogeneity of HTA practices across jurisdictions.

The framework presented here is a core evaluation framework; it contains the full spectrum of value domains that are—based on the current state of the literature—internationally relevant. The core framework is proposed to be the starting point for developing decision context-specific value assessment frameworks, hence it cannot be used in its current format, but should be adapted to national settings. It is also important to highlight that each individual VAM is not expected to provide benefits in all value domains. This is illustrated in Additional file 1: Table S3, where example cases (described in Additional file 1: Table S1) are presented alongside the value domains of the framework, showing in which value domains they offer additional benefits compared to standard care.

Since some value domains may not be equally relevant or accepted in all countries, the proposed core framework needs thorough adaptation in all jurisdictions. Similarly, the value domains in the proposed framework are not ranked based on their relevance. Therefore, the relative importance and scoring rules of domains shall be determined during future national adaptation(s). During the development of scoring rules, one of the fundamental challenges is to determine the cut-off point (i.e., the magnitude of the difference in certain value attributes), which is perceived as a meaningful benefit and is rewarded [29]. Although decision-makers in different countries might prioritize different value domains in their own jurisdiction, international consensus on the application of a core evaluation framework could provide guidance to manufacturers on which types of benefits are potentially acknowledged by national payer bodies.

Even though the authors followed a rigorous methodology during evidence synthesis and the development of the framework, the presented research is subject to potential limitations. First, value propositions were solely extracted from literature published before the database search had been conducted. Therefore, to capture benefits delivered by future directions of evolutionary innovation (e.g., environmental sustainability of health technologies), the core evaluation framework should be reviewed and updated periodically [30]. Furthermore, although experts invited for external validation represented countries with different economic statuses and decision-making contexts, their number was relatively small. They were identified in an iterative process by exploiting the professional network of the research team.

Even though efforts were made during the internal validation procedure to minimize overlaps and interaction between the value domains, these could not be eliminated entirely due to interdependence and issues of causality. For example, side effects or patient satisfaction can influence the adherence of patients, which can eventually influence the real-world effectiveness of medicines. Further research is needed to explore and determine the extent of potential interrelation among the proposed domains. Additionally, since the presented framework is a core framework, determining importance (i.e., the final selection of domains and elicitation of their relative weights) and scoring functions of the domains should be done by further research during the national adaptations. After national adaptation(s), once the framework is pilot tested in real policy decisions, it should be re-iterated based on the accumulated experience before using it routinely for the evaluation of VAMs. Such revisions may be implemented with a greater pool of decision-makers. The abovementioned steps could further increase the local relevance of the framework.

## Conclusion

The core framework proposed in this paper captures the health care and societal benefits of VAMs and provides a starting point for national decision-makers, who are willing to differentiate VAMs from originator medicines and generics. As the framework can be adapted to different decision-making contexts, it has the potential to reduce the heterogeneity in value assessment processes across countries and consequently create incentives for manufacturers to invest into further improving medicinal products. Finally, the presented framework calls for a paradigm shift in how incremental innovation could be approached by policy-makers. This would facilitate the timely market entry of pharmaceutical products that could improve the sustainability of health systems globally, increase patients' access to affordable and much needed patient-centered therapies and address emerging public health concerns.

## Supplementary Information

**Additional file 1**: **Table S1**. Example cases for the different repurposing models (based on the IQVIA report) [17]. **Table S2.** Search syntax for the systematic literature review. **Table S3.** Illustrative benefits of value added medicine test cases according to the domains of the core evaluation framework as reported by the IQVIA report [17]. **Table S4.** A non-exhaustive list of value propositions in each value domain.

## Data Availability

All data generated or analysed during this study are included in this published article [and its Additional files].

## Abbreviations

HTA: Health technology assessment
MCDA: Multi-criteria decision analysis
MFE: Medicines for Europe
SLR: Systematic literature review
TLR: Targeted literature review
VAM: Value-added medicines
