# Supplementary material for: Development of a core evaluation framework of value-added medicines: report 1 on methodology and findings
Source: Cost Eff Resour Alloc. 2021 Aug 31;19:57. doi: 10.1186/s12962-021-00311-6 (PMC8406013; doi:10.1186/s12962-021-00311-6)
Supplement: Supplementary file 1 — Additional file 1: Table S1. Example cases for the different repurposing models (based on the IQVIA report) [17]. Table S2. Search syntax for the systematic literature review. Table S3. Illustrative benefits of value added medicine test cases according to the domains of the core evaluation framework as reported by the IQVIA report [17]. Table S4. A non-exhaustive list of value propositions in each value domain. [file 12962_2021_311_MOESM1_ESM.docx]

Supplementary Table 1 - Example cases for the different repurposing models (based on the IQVIA report) [17]

| **Repurposing model** | **Example case** | **Description** | **Main value proposition** |
| --- | --- | --- | --- |
| Repositioning | Guanfacine | - Originally used in hypertension - Repositioned to ADHD | Guanfacine offers an important alternative “non-stimulant” therapeutic option for patients who cannot tolerate stimulant type medication |
| Reformulation | Paclitaxel | - Used in oncology - Reformulated in an albumin-bound nanoparticle | Enhanced tissue distribution of reformulated paclitaxel resulted in:   - reduction in treatment time from 3 hours to 30 mins, - elimination of the need for pre-medication with steroids and antihistamines and - improvement in oncologic response rates and overall survival |
| Combination  (2 oral active compounds) | Oxycodon + Naloxone | - Used to alleviate moderate to severe pain - Oral combination of 2 off-patent compounds | Oxycodone treats the pain while naloxone counteracts the opioid-induced constipation (common and severe side-effect) |
| Combination  (with new inhaler) | Budesonide + Formoterol +  new device | - Used in COPD - Combination with a new inhaler device | New inhaler reduces common inhaler preparation errors and allows patients to use the inhaler while lying down |

(ADHD - Attention Deficit Hyperactivity Disorder, COPD - Chronic Obstructive Pulmonary Disease)

Supplementary Table 2 - Search syntax for the systematic literature review

| **Set** | **Search Terms** |
| --- | --- |
| 1 | ("added value" OR "me too" OR "value added" OR added-value OR bio-better OR value-added OR repurpos*) |
| 2 | (drug[Title/Abstract] OR drugs[Title/Abstract] OR generic[Title/Abstract] OR generics[Title/Abstract] OR medicat*[Title/Abstract] OR medicin*[Title/Abstract] OR pharmaceutical[Title/Abstract] OR pharmaceuticals[Title/Abstract] OR product[Title/Abstract] OR products[Title/Abstract] OR therap*[Title/Abstract]) |
| 3 | ("adaptive innovation" OR "drug rediscovery" OR "drug reformulation" OR "drug repositioning" OR "drug reprofiling" OR "drug re-profiling" OR "drug repurposing" OR "incremental innovation" OR "marginal innovation" OR "specialty generics" OR "super generics" OR "adapted generics" OR "customized generics" OR "tailored generics") |
| 4 | ("value attributes" OR "value drivers" OR "value messages" OR "value proposition" OR "value propositions" OR "value attribute" OR "value driver" OR "value message" OR advantage OR advantages OR benefit OR benefits OR "savings" OR "cost-saving" OR "cost saving" OR "cost reduction" OR "cost-reduction" OR "reduction of costs" OR "multi-criteria decision analysis" OR MCDA OR "multiple criteria decision aiding" OR "multi-criteria decision making" OR "MCDM" OR "multi-criteria analysis" OR "multi-attribute decision analysis" OR "value framework" OR "value assessment") |
|  | (((#1 AND #2) OR #3) AND #4) |

Supplementary Table 3 – Illustrative benefits of value added medicine test cases according to the domains of the core evaluation framework as reported by the IQVIA report [17]

| **Example case** | Repurposing model | Extending treatment options in new indication with unmet medical need | Individual needs/special needs of patient (sub)population | Efficacy/ Effectiveness | Patient safety and  tolerability | Patient experience related to the therapy | Adherence and Persistence | Quality of life | Patient’s economic burden | Economic and health burden on informal caregiver | Health care resource utilization, costs or efficiency | Technological improvement with logistical considerations |
| --- | --- | --- | --- | --- | --- | --- | --- | --- | --- | --- | --- | --- |
| ***guanfacine*** | repositioned  to ADHD | **✔** |  |  |  | **✔** | **✔** |  |  |  |  |  |
| ***paclitaxel*** | reformulation |  |  | **✔** |  |  |  |  | **✔** |  | **✔** |  |
| ***oxycodon +naloxone*** | oral combination |  |  |  | **✔** | **✔** | **✔** |  |  |  | **✔** |  |
| ***budesonide +formoterol +device*** | combination with a new inhaler |  | **✔** |  |  | **✔** | **✔** |  |  |  |  |  |

(ADHD - Attention Deficit Hyperactivity Disorder)
**✔** Checkmarks indicate the value domains in which the example products offer additional value

Supplementary Table 4 – A non-exhaustive list of value propositions in each value domain

| **Domain name** | **Value propositions (non-exhaustive examples)** |
| --- | --- |
| Extending treatment options in new indication with unmet medical need | - provides greater choice to doctors - limit off-label use of medicines - provide new treatment option with orphan designation (medicine previously used in a common disease) |
| Individual needs/special needs of patient (sub)population | - provides greater personalization - increased number of available dosing options - tailor therapies to specific patient populations - new treatment option for drug resistant patients |
| Efficacy/Effectiveness | - increased OS/PFS - fewer exacerbations - decreased mortality - delay disease progression - reducing relapse events |
| Patient safety and tolerability | - improved tolerability - less side-effects - therapeutic dose-monitoring to avoid toxicity - better on-site dose, with dose reduction |
| Patient experience related to the therapy | - greater ease of use - reduced pill burden - improved taste - painless and comfortable application |
| Adherence and Persistence | - improved compliance - reduce medication errors - sustained drug release with longer treatment effect - enhanced persistence |
| Quality of life | - improvement of well-being and symptoms - improved functionality - greater mobility - better mental state - less pain |
| Patient's economic burden | - shorter self-administration time - reduced number of missed days at work - enhanced work productivity |
| Economic and health burden on informal caregiver | - decreased treatment burden for caregiver - less associated out-of-pocket cost of care - less patient supervision is required |
| Health care resource utilization, costs or efficiency | - less associated cost for the healthcare system - positive impact on patient pathway - reduce drug wastage |
| Technological improvement with logistical considerations | - improved product stability - medicine does not require special storage conditions - improved shelf life |
